# Interleukin (IL)-1β Is a Strong Inducer of IL-36γ Expression in Human Colonic Myofibroblasts

**DOI:** 10.1371/journal.pone.0138423

**Published:** 2015-11-12

**Authors:** Kenichiro Takahashi, Atsushi Nishida, Makoto Shioya, Hirotsugu Imaeda, Shigeki Bamba, Osamu Inatomi, Tomoharu Shimizu, Katsuyuki Kitoh, Akira Andoh

**Affiliations:** 1 Department of Medicine, Shiga University of Medical Science, Seta-Tukinowa, Otsu 520–2192, Japan; 2 Department of Surgery, Shiga University of Medical Science, Seta-Tukinowa, Otsu 520–2192, Japan; Temple University School of Medicine, UNITED STATES

## Abstract

**Backgrounds and aims:**

Interleukin (IL)-36 cytokines are members of the IL-1 cytokine family. In this study, we investigated the expression of IL-36γ in human colonic myofibroblasts to explore the molecular mechanisms underlying IL-36γ induction.

**Materials and methods:**

IL-36 mRNA was analyzed by real-time PCR method. Secretion of IL-36γ protein was evaluated by Western blot and ELISA analyses. Molecular mechanism of IL-36γ induction was evaluated by siRNA analyses and immunofluorescence experiments.

**Results:**

IL-36γ mRNA expression was scarcely detected in the cells without stimulation. IL-1β induced a marked increase of IL-36γ mRNA expression. TNF-α markedly enhanced IL-1β-induced IL-36γ mRNA expression. These responses were confirmed at the protein levels. The inhibitors for ERK1/2 (PD98059 and U0216) and a p38 MAPK (SB203580) significantly reduced the IL-1β-induced IL-36γ mRNA expression. In addition, the siRNAs specific for NF-κB p65 and AP-1 (c-Jun) significantly reduced the expression of IL-1β-induced IL-36γ mRNA.

**Conclusions:**

Colonic myofibroblasts are cellular source of IL-36γ in the intestine. IL-36γ expression was induced by the combination of IL-1β and TNF-α via activation of MAPKs and transcription factors, NF-κB and AP-1.

## Introduction

Inflammatory bowel diseases (IBD), ulcerative colitis (UC) and Crohn’s disease [1], are chronic intestinal disorders of unknown etiology. The pathogenesis of IBD is thought to be an aberrant response of the mucosal immune system towards luminal antigens such as dietary factors and/or commensal bacteria in genetically susceptible individuals [2–5]. IBD is often characterized by an imbalance between the effector and the regulatory activities of intestinal immunity, with a preponderance of proinflammatory cytokines [6, 7].

IL-36α (previously known as IL-1F6), IL-36β (IL-1F8) and IL-36γ (IL-1F9) are recently reported members of the IL-1 cytokine family [8–10]. IL-36 cytokines have been identified initially through use of DNA data base searches for homologs to IL-1 [11]. They were classified as IL-1 family members based on amino acid sequence similarity, identity of gene structure, and predicted or known three-dimensional structure [11]. Each member of the IL-36 cytokines activates NF-κB and MAPK pathways [8, 11] via binding to a heterodimeric receptor consisting of the IL-36 receptor (IL-36R) subunit and the IL-1 receptor accessory protein (IL-1RAcP) [11, 12]. Recent studies showed that IL-36 cytokines contribute to the pathophysiology of chronic inflammatory disorders such as psoriasis [13–15], rheumatoid arthritis [16, 17] and pulmonary disease [18, 19]. However, there are no known reports regarding the association between IL-36 cytokines and chronic intestinal inflammation. Moreover, regulatory mechanisms underlying IL-36 induction remain unclear in any cell types.

In this study, we investigated the expression of IL-36γ in human colonic myofibroblasts to explore the mechanisms underlying IL-36γ induction. Colonic myofibroblasts are located subjacent to the basement membrane of the intestinal mucosa [20], and play critical roles in the pathophysiological processes involved in inflammation and mucosal healing in the intestine [20, 21]. To our knowledge, this is the first study demonstrating IL-36γ induction from the cells resident in the intestine and molecular mechanisms of IL-36γ induction. These responses suggest that IL-36γ might play an important role in the pathophysiology of gut inflammation.

## Materials and Methods

### Reagents

Recombinant human cytokines and anti-human IL-36γ were purchased from R&D Systems (Minneapolis, MN). Inhibitors of p42/44 MAPK (PD98059 and U0216) and inhibitor for p38 MAPK (SB203580) were purchased from Merck (Darmstadt, Germany). siRNA for NF-κB p65, c-Jun and a control siRNA were purchased from Santa Cruz (Santa Cruz, CA). Antibodies against phosphorylated and total p42/44 MAPK (ERK1/2), p38 MAPK, JNK1/2, GAPDH and laminin A/C were purchased from Cell Signaling Technology (Beverly, MA). Antibodies against phosphorylated c-Jun, NF-κB p65, phosphorylated IκBα were purchased from Santa Cruz. All other reagents were purchased from Sigma Chemical Co. (St Louis, MO).

### Culture of human colonic subepithelial myofibroblasts

The primary culture of human colonic myofibroblasts was prepared according to the method reported by Mahida et al [22]. The cellular characteristics and culture conditions have been described in our previous report [23]. Samples of the human adult colonic mucosa were obtained from healthy part of surgical specimens (>5 cm from the tumor margin) of patients undergoing a partial colectomy for carcinoma. The purity of subepithelial myofibroblastswas was confirmed by α-smooth muscle actin-positivity, and was above 95%. All studies were performed on passages 3–6 of myofibroblasts isolated from six resection specimens. The ethics committee of the Shiga University of Medical Science approved this project, and written informed consent was obtained from all patients.

### Immunocytochemistory for c-Jun and NF-κB p65

Cells were grown on a culture slide system (BD, Franklin Lakes, NJ), fixed with 4% paraformaldehyde and reacted with anti-phosphorylated c-Jun and anti-NF-κB p65 antibodies. Then, they were incubated with fluorescence-labeled second antibodies. Nuclei were visualized using mounting medium with DAPI (Vector Laboratories, Burlingame, CA). A digital confocal laser scanning microscope (Nikon, Tokyo, Japan) was used for analysis.

### Quantification of human IL-36 cytokines mRNA

The mRNA expression of IL-36 cytokines was assessed by reverse-transcription (RT)-PCR and real-time PCR analyses. The oligonucleotide primers used in this study were IL-36γ (sense: GTCTATCAATCAATGTGTAAACC, anti-sense: ATCTTCTG- CTCTTTTAGCTGCAAT) [24] and β-actin (sense: TGACCCAGATCATGT- TTGAGACCT, anti-sense: CCACGTCACACTTCATGATGATGGAG). The real-time PCR was performed using a Light Cycler 480 system (Roche Applied Science). The PCR was performed using a SYBR Premix Ex Taq (TAKARA, Japan). The data were normalized versus β-actin for human IL-36γ.

### ELISA for IL-36γ

IL-36γ protein in the cell culture supernatant was quantified by an ELISA kit, purchased from Uscn Life Science (Wuhan, China).

### Western blot analysis

The cells were lysed in a SDS sample buffer containing orthovanadate. Western blot was performed according to a method previously described [25]. The detection was performed using the enhanced chemiluminescence Western blotting system (GE Healthcare, Little Chalfont, UK). Nuclear proteins were extracted using the CelLytic NuCLEAR Extraction Kit (Sigma-Aldrich, St. Louis, MO) and were subjected to Western blot analyses.

### NF-κB p65 and c-Jun mRNA interference (RNAi) experiments

siRNA duplex for NF-κB p65 and c-Jun was mixed with Lipofectamine™ RNAi MAX Reagent (Invitrogen, Carlsbad, CA) in Opti-MEM™ medium and incubated with the cells for 60 hours.

### Statistical Analysis

The statistical significance of the differences was determined by Student`s t- test (GraphPad Prism 6.0; GraphPad Software, Inc., La Jolla, CA). One-way analysis of variance (one-way ANOVA) with Bonferroni post-test (GraphPad Prism 6.0; GraphPad Software, Inc.) was used in dose- and time-dependent study. Differences resulting in P values less than 0.05 were considered to be statistically significant.

## Results

### IL-36γ expression in human colonic myofibroblasts

**Fig 1:**
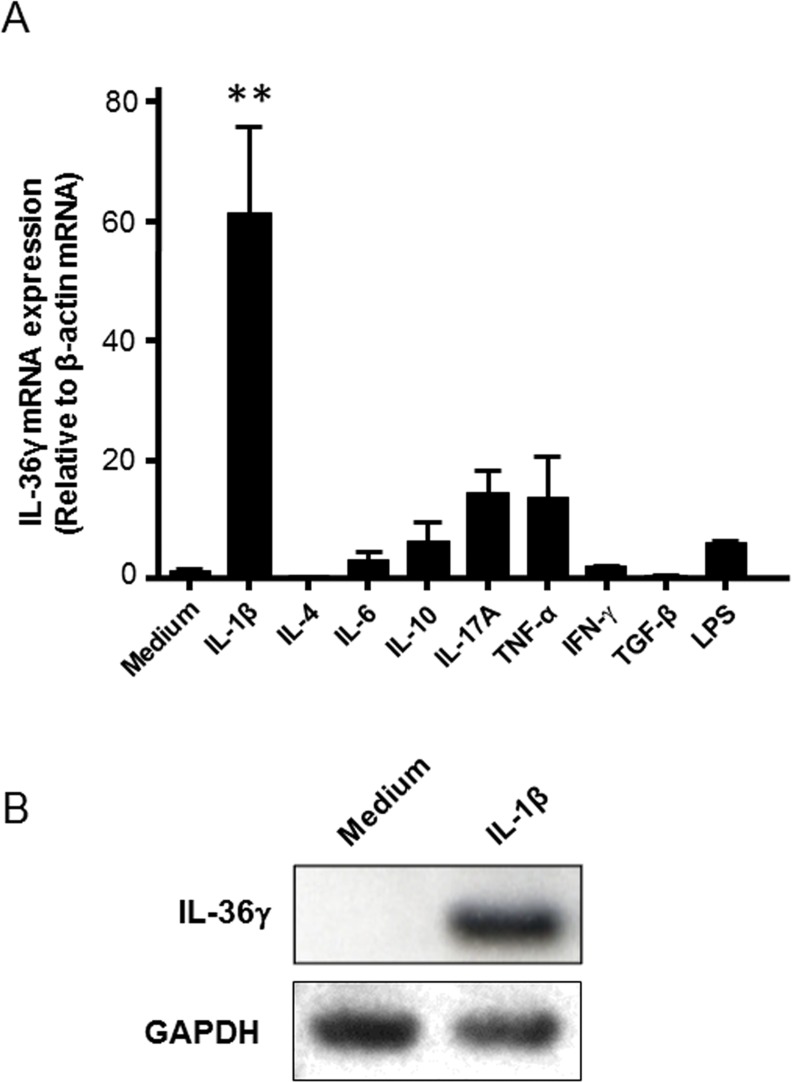
IL-36γ mRNA and protein expression in human colonic myofibroblasts. (A) IL-36γ mRNA expression in human colonic myofibroblasts. The cells were stimulated with cytokines (100 ng/ml) for 24h, and IL- 36γ mRNA expression was analyzed by real-time PCR. IL-36γ mRNA expression was expressed as relative to β-actin mRNA expression (mean ± SD from 4 different experiments). **P < 0.01 versus medium only. (B) IL-36γ protein expression. The cells were stimulated with or without IL-1β (10 ng/ml) for 24h, and intracellular IL-36γ was analyzed by Western blot.

IL-36γ mRNA expression was investigated in human colonic myofibroblasts. The cells were cultured for 24h in the absence or presence of cytokines (100 ng/ml), and IL-36γ mRNA expression was analyzed by real-time PCR. As shown in Fig 1A, IL-36γ mRNA expression was scarcely detected in the cells cultured in medium alone. A marked and significant increase of IL-36γ mRNA expression was induced by IL-1β. Western blot analyses showed intracellular IL-36γ protein expression in response to IL-1β stimulation (Fig 1B).

### IL-36γ induction by IL-1β

**Fig 2:**
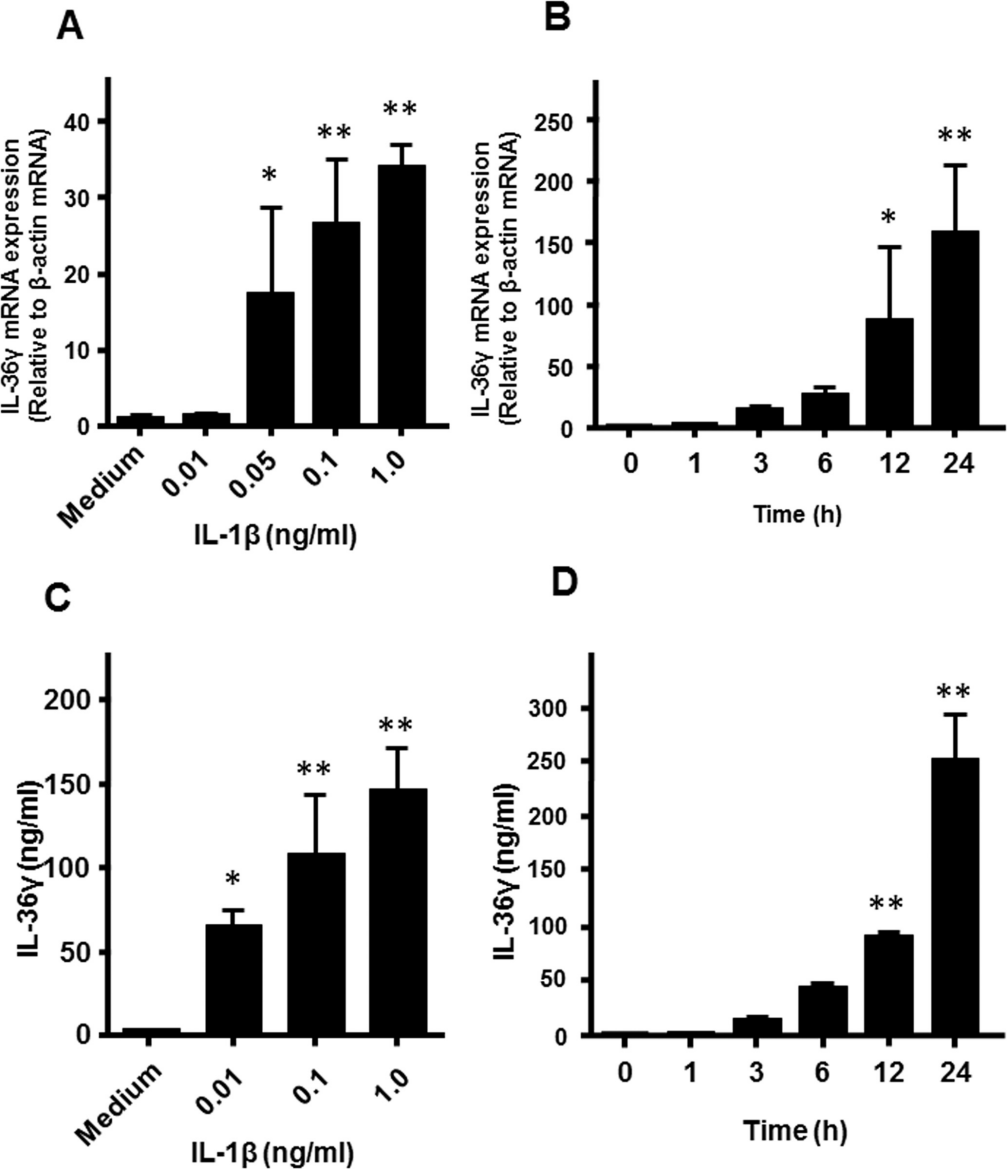
Effects of IL-1β on IL-36γ mRNA expression in colonic myofibroblasts. (A) Dose-dependent effects of IL-1β on IL-36γ mRNA expression. The cells were incubated for 24 h with increasing concentrations of IL-1β. IL-36γ mRNA expression was expressed relative to β-actin mRNA expression (mean ± SD from 4 different experiments). (B) Time-dependent effects of IL-1β on IL-36γ mRNA expression. The cells were stimulated with IL-1β (10 ng/ml) for the pre-determined times. IL-36γ mRNA expression was expressed relative to β-actin mRNA expression (mean ± SD from 4 different experiments). (C) Dose-dependent effects of IL-1β on IL-36γ secretion. The cells were incubated for 24 h with increasing concentrations of IL-1β. IL-36γ level in supernatant was determined by ELISA (mean ± SD from 4 different experiments). (D) Time-dependent effects of IL-1β on IL-36γ secretion. The cells were stimulated with IL-1β (10 ng/ml) for the pre-determined times. IL-36γ level was determined by ELISA (mean ± SD from 4 different experiments). *P<0.05, **P<0.01 versus medium; ANOVA followed by Bonferroni’s post hoc test.

Colonic myofibroblasts were stimulated for 24h with increasing concentrations of IL-1β, and expression of IL-36γ mRNA was determined by real-time PCR. As shown in Fig 2A, IL-1β dose-dependently induced IL-36γ mRNA expression, and a significant effect was detected at 0.05 ng/ml of IL-1β. Furthermore, the kinetics of IL-36γ mRNA induction in response to IL-1β was analyzed. A significant induction of IL-36γ mRNA was detected as early as 3h after IL-1β stimulation, and then IL-36γ mRNA expression was time-dependently induced (Fig 2B).

The effects of IL-1βwere evaluated at the protein level. Cells were stimulated for 24h with increasing concentrations of IL-1β, and secreted IL-36γ levels were determined by ELISA. As shown in Fig 2C, IL-1β dose-dependently induced IL-36γsecretion. IL-1β also induced time-dependent secretion of IL-36γ (Fig 2D).

### Combined effects of cytokines on IL-36γ mRNA expression

**Fig 3:**
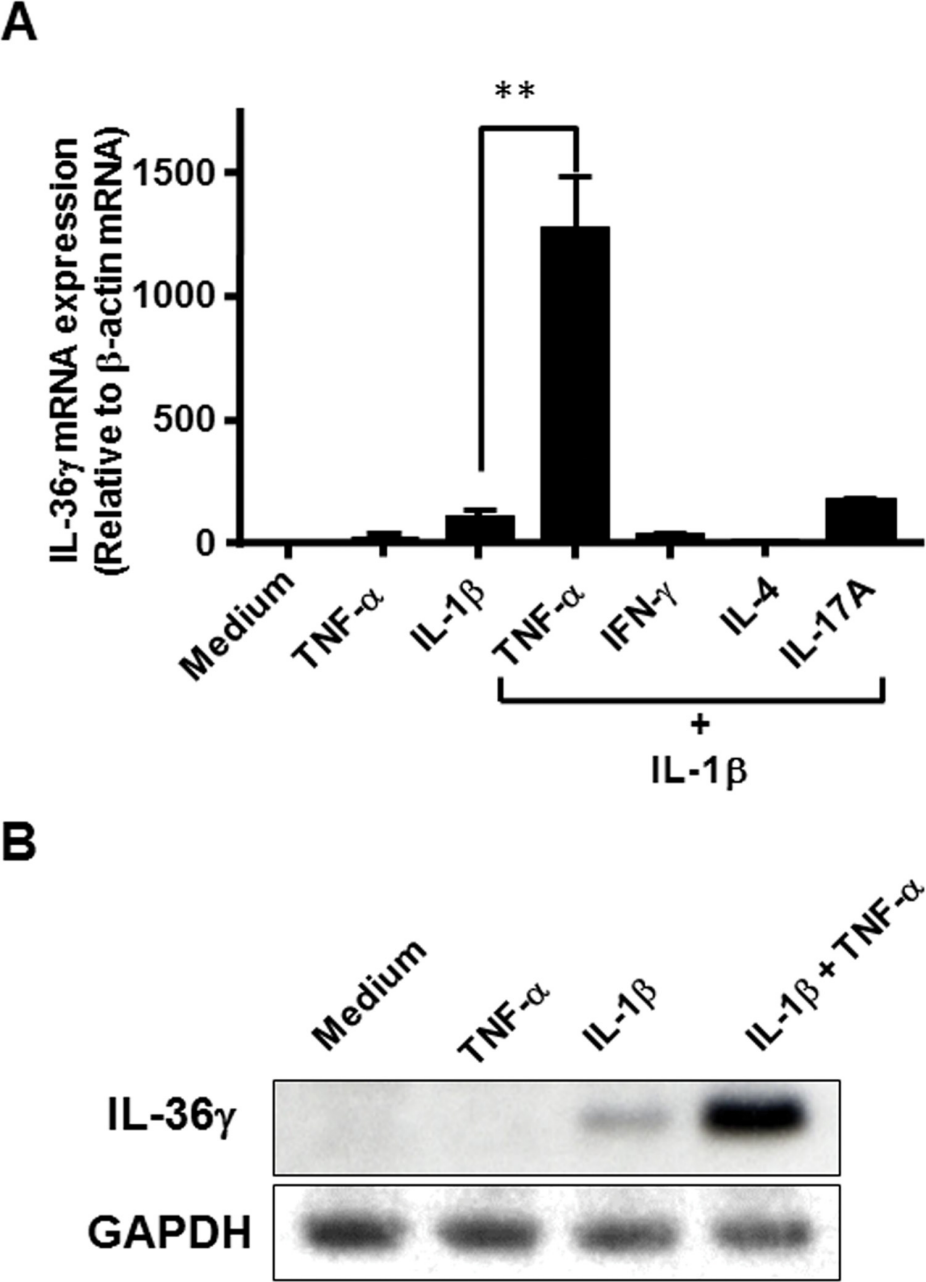
Combined effects of IL-1β and other cytokines. (A) The cells were incubated for 24 h with combination of IL-1β (10 ng/ml) and other cytokines [TNF-α (100 ng/ml), IFN-γ (100 ng/ml), IL-4 (100 ng/ml), and/or IL-17A (100 ng/ml)]. IL-36γ mRNA expression was analyzed by real-time PCR. IL-36γ mRNA expression was expressed relative to the β-actin mRNA expression (mean ± SD from 4 different experiments). **P<0.01 versus IL-1β alone. (B) The cells were stimulated for 24 h with or without IL-1β (10 ng/ml), TNF-α (100 ng/ml), and/or combination of IL-1β (10 ng/ml) and TNF-α (100 ng/ml), and intracellular IL-36γ was analyzed by Western blot.

Colonic myofibroblasts were simulated for 24h with combination of IL-1β (10 ng/ml) plus various cytokines (100ng/ml). As shown in Fig 3A, simultaneous stimulation with IL-1β and TNF-α showed a synergistic increase of IL-36γ mRNA expression. This was also confirmed at the protein level by Western blot analysis (Fig 3B).

### Role of MAPK activation in IL-1β-induced IL-36γ mRNA expression

**Fig 4:**
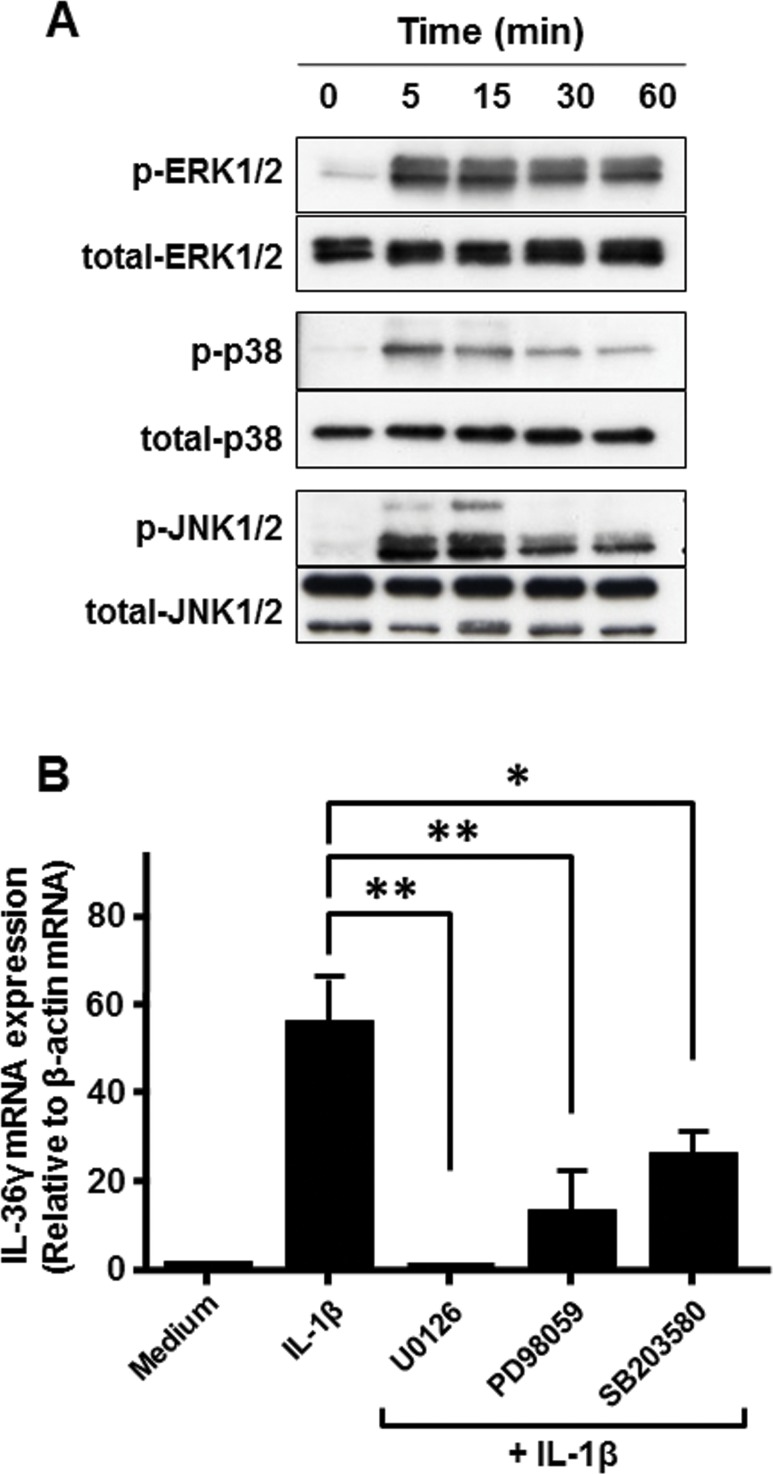
Involvement of MAPK activation in IL-1β-induced IL-36γ expression. (A) MAPK activation in colonic SEMFs. The cells were stimulated with IL-1β (10 ng/ml), and the activation of MAPKs were analyzed by Western blotting. Antibodies against phosphorylated (p)- and total- MAPKs were used. *P < 0.05, **P<0.01. (B) The cells were stimulated for 24 h with IL-1β (10ng/ml) in the presence or absence of MEK inhibitors [U0216 (10 μM) and PD98059 (10 μM)] and a p38 inhibitor [SB203580 (10 μM)]. IL-36γ mRNA expression was analyzed by real-time PCR. IL-36γ mRNA expression was expressed relative to the β-actin mRNA expression (mean ± SD from 4 different experiments).

To investigate the molecular mechanisms underlying IL-1β-induced IL-36γ induction in colonic myofibroblasts, we evaluated whether the activation of MAPKs are involved in the IL-1β-induced IL-36γ mRNA expression. As shown in Fig 4A, IL-1β (10ng/ml) induced a phosphorylation of ERK1/2, p38 MAPK, and JNK1/2 as early as 5 min after stimulation. The addition of inhibitors for ERK1/2 (PD98059 and U0216) and a p38 MAPK inhibitor (SB203580) significantly reduced IL-1β-induced IL-36γ mRNA expression (Fig 4B). These results indicated that IL-1β-induced IL-36γ induction was mediated by activation of ERK1/2 and p38 MAPKs.

### IL-1β-induced IL-36γ mRNA expression is mediated by activation of transcription factors NF-κB and AP-1

**Fig 5:**
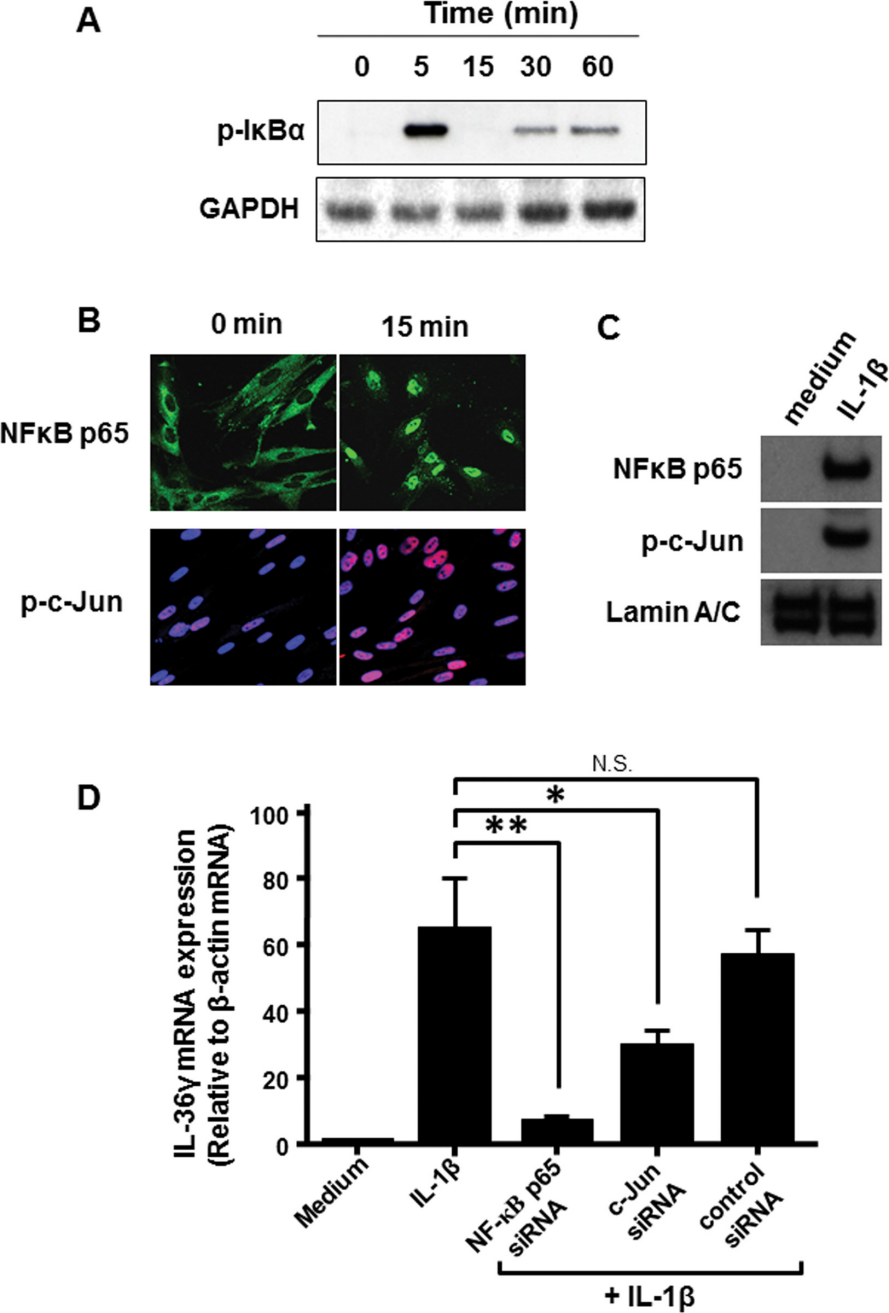
Involvement of activation of transcription factors, NF-κB and /AP-1, in IL-1β-induced IL-36γ expression. (A) IL-1β induced IκBα phosphorylation and degradation in colonic myofibroblasts. The cells were stimulated with IL-1β (10 ng/ml), and phosphorylated IκBα were detected by Western blotting. (B) IL-1β induced activation of NF-κB and c-Jun AP-1. The cells were stimulated with IL-1β (10 ng/ml), and NF-κB p65 and phosphorylated c-Jun were detected by immunocytochemistory. Reacted antibodies against NF-κB p65 were visualized by FITC (green fluorescence)-labeled second antibody. Reacted antibodies against phosphorylated c-Jun were detected by a DyLight^®^ 594 (red fluorescence)-labeled secondary antibodies. Nucleus was stained by DAPI (blue). (C) The cells were stimulated with IL-1β (10 ng/ml) or medium alone for 15 min and nuclear proteins were extracted. NF-κB p65 and phosphorylated c-Jun in nuclear extracts were detected by immunoblot. (D) Effects of silencing of NF-κB p65 and c-Jun AP-1on IL-1β-induced IL-36γ expression. The cells were transfected with control siRNA, the siRNA specific for NF-κB p65 and/or c-Jun AP-1, and incubated for 24h. IL-36γ mRNA expression was analyzed by real-time PCR. IL-36γ mRNA expression was expressed relative to the β-actin mRNA expression (mean ± SD from 4 different experiments). *P<0.05, **P < 0.01 versus IL-1β stimulation.

Next, we assessed the role of the transcriptional factors NF-κB and AP-1 in the IL-1β-induced IL-36γ mRNA expression. Activation of the NF-κB is initiated by the signal-induced degradation of IκB proteins by the proteasome [26]. The activated NF-κB is then translocated into the nucleus where it binds to specific sequences of DNA [26]. In colonic myofibroblasts, Western blot analysis showed that IL-1β rapidly induced a phosphorylation of IκBα within 5 min, and this was completely degraded 15 min after IL-1β stimulation (Fig 5A). As shown in Fig 5B, immunohistochemistry showed that NF-κB p65 was located in the cytosol in inactivated cells, and this was translocated into the nucleus 15 min after IL-1β stimulation. This was confirmed by Western blot for NF-κB p65 of nuclear extracts (Fig 5C). These indicate NF-κB activation in response to IL-1β in colonic myofibroblasts. The AP-1 (c-Jun) activation was also analyzed by immunohistochemical procedure. As shown in Fig 5B, phosphorylated c-Jun was detected as pink fluorescence 15 min after IL-1β stimulation (blue color indicates nuclear staining). In addition, Western blot analysis using nuclear extracts showed a rapid accumulation of phosphorylated c-Jun into the nucleus (Fig 5C). This indicates AP-1 (c-Jun) activation in response to IL-1β. These findings are supported by our previous experiments of EMSA (electrophoretic mobility shift assay) that IL-1β enhanced NF-κB- and AP-1-DNA binding activity in colonic myofibloblasts [27].

Based on these findings, we evaluated the effects of siRNAs specific for NF-κB p65 and AP-1 (c-Jun) on IL-1β-induced IL-36γ mRNA expression. As shown in Fig 5D, the control siRNA revealed no effect, and the silencing of NF-κB p65 and AP-1 (c-jun) significantly reduced the expression of IL-1β-induced IL-36γ mRNA. Collectively, these findings indicate that IL-1β-induced IL-36γ expression is mediated by the activation of transcriptional factors, NF-κB and AP-1.

## Discussion

IL-36 cytokines are reported to be involved in the pathogenesis of chronic inflammatory disorders such as psoriasis, lung inflammation, and rheumatoid arthritis [13, 15, 16]. However, there are no reports regarding the role of IL-36 cytokines in chronic intestinal inflammation, including IBD. Moreover, the molecular mechanism responsible for IL-36γ induction has not been identified in any cell types. To our knowledge, the current study is the first report concerning IL-36γ expression from the primary cultured cells isolated from human intestinal mucosa and its regulatory mechanism.

IL-36α expression was shown by a variety of cells, including keratinocytes, T and B lymphocytes as well as monocytes in the lesions of rheumatoid arthritis and psoriasis [17, 28]. However, there is no report of IL-36γ expression in human colonic tissues. Colonic myofibroblasts have been reported to play a critical role in the gut inflammatory and regenerative responses through the expression of various active mediators including cytokines [20]. This leads us to investigate IL-36γ expression in these cells. Among various cytokines, we found that IL-1β is a strong inducer of IL-36γ in colonic myofibroblasts. TNF-α has been reported to induce IL-36γ in bronchial epithelial cells [19], but showed a minimal effect in colonic myofibroblasts. Interestingly, combination of IL-1β plus TNF-α exerted a remarkable effect on IL-36γ expression. These findings indicate that IL-36γ expression is mainly mediated by IL-1β and that TNF-α is also an important factor through synergistic effect with IL-1β. Collectively, colonic myofibroblasts are cellular source of IL-36γ in the intestinal mucosa, and two major proinflammatory cytokines, IL-1β and TNF-α, play a pivotal role in the induction of IL-36γ.

Molecular mechanisms underlying IL-36γ induction have not fully been identified in any cell types. At first, we investigated involvement of MAPKs in IL-1β-induced IL-36γ expression. MAPK activation has been reported as an important signaling event in response to proinflammatory stimuli [29]. Three subgroups of the MAPK family (the p44 and p42 ERK1/2, the p38 MAPK and the c-Jun NH2-terminal kinase) have been identified. These are phosphorylated on activation by upstream kinases, the MAPK kinases [29]. In this study, IL-1β induced a rapid activation of MAPKs in colonic myofibroblasts, and the role of the MAPKs in IL-1β-induced IL-36γ expression was assessed by using specific inhibitors. Imidazole compound SB203580 is a specific inhibitor of p38 MAPK [30], and induced a significant decrease in the IL-1β-induced IL-36γ mRNA expression. We also addressed the role of ERK1/2 in our system. PD98059 is a specific inhibitor of MAPK/ERK kinase (MEK1) [31], the kinase directly upstream to ERK1/2, and U0126 is a specific inhibitor of MEK1 and MEK2 [32]. PD98059 and U0126 caused a significant inhibition of the IL-1β-induced IL-36γ expression. Thus, we concluded that p38 and ERK1/2 MAPKs participate in the IL-36γ expression induced by IL-1β in human colonic myofibroblasts.

Many cytokine-inducible responses are mediated by DNA-binding proteins such as NF-κB [33]. IL-36 cytokines have been reported to activate NF-κB pathway [8, 11] via binding to a heterodimeric receptor consisting of IL-36R and IL-1RAcP [11, 12]. However, the role of NF-κB pathway in IL-36 induction has not been investigated previously. We demonstrated for the first time that the activation of NF-κB was necessary for IL-1β-induced IL-36γ expression in human colonic myofibroblasts. Evidences supporting this can be summarized. First, IL-1β rapidly induced a rapid activation of IκBα and translocation of NF-κB p65 into the nucleus. Second, inhibition of NF-κB activation by siRNA induced a marked decrease in IL-1β-induced IL-36γ expression. Furthermore, we also found a critical role of c-Jun AP-1 in IL-1β-induced IL-36γ expression in colonic myofibroblasts. The activation of c-Jun AP-1 was induced by IL-1β stimulation and blocking of c-Jun using siRNA significantly reduced the IL-1β-induced IL-36γ expression. As a limitation, we could not show direct evidence that IL-1β induced the binding of NF-κB and AP-1 to the IL-36γ promoter region. So, possible contribution of other mechanisms, such as MAPKs-mediated post-transcriptional mechanism reported in IL-8 mRNA expression [34], should be investigated in the future. Taken together, it was suggested that NF-κB and AP-1 play a critical role in IL-1β-induced IL-36γ expression in colonic myofibroblasts.

The function of IL-36 cytokines in intestinal inflammatory disorders still remains unclear. It is well recognized that increased T cell responses play a critical role in the pathophysiology of chronic inflammatory disorders [3]. Vigne et al. reported that IL-36R was predominantly expressed on naïve CD4^+^ T cells and that IL-36 cytokines directly stimulates proliferation and IL-2 secretion of T cells [35]. IL-36 signaling directly promoted Th1 polarization of naïve CD4^+^ T cells [35] and induced Th17 immune response [36]. Mutamba et al. have reported that IL-36γ stimulates maturation of monocyte-derived dendritic cells [37]. In addition, preliminary experiment in our laboratory showed that IL-36 stimulates chemokine secretion from colonic myofibroblasts, suggesting IL-36-mediated autocrine loop of inflammation in colonic myofibroblasts. At present, there are no known reports regarding the association between IL-36 and chronic intestinal inflammation. However, accumulating data suggest an involvement of IL-36 in the pathophysiology of chronic intestinal inflammation.

In conclusion, we showed IL-36γ secretion from human colonic myofibroblasts. In addition, we found that IL-1β is a strong inducer of IL-36γ expression via MAPK pathways and NF-κB/AP-1 activation.
