# Expression of Concern: Interleukin (IL)-1β Is a Strong Inducer of IL-36γ Expression in Human Colonic Myofibroblasts

**DOI:** 10.1371/journal.pone.0279488

**Published:** 2023-01-11

**Authors:** The PLOS ONE Editors

The Funding Statement for this article [1] states that the study received funding from the Smoking Research Foundation, which according to [2] has received financial support from the tobacco industry. In light of this issue the *PLOS ONE* article [1] does not comply with the journal’s policy on Funding from Tobacco Companies [3] which was implemented in 2010.

Therefore, the *PLOS ONE* Editors issue this Expression of Concern.

In post-publication discussions the corresponding author stated that Smoking Research Foundation funding did not support this study and was reported in error.

​​​​We regret that this concern was not identified and addressed prior to the article’s [1] publication.
